# Conditional Vitamin D Receptor Deletion Induces Fungal and Archaeal Dysbiosis and Altered Metabolites

**DOI:** 10.3390/metabo14010032

**Published:** 2024-01-01

**Authors:** Duncan J. Claypool, Yong-Guo Zhang, Yinglin Xia, Jun Sun

**Affiliations:** 1 Department of Medicine, University of Illinois Chicago, Chicago, IL 60612, USA; dclayp2@uic.edu (D.J.C.); yongguo@uic.edu (Y.-G.Z.); 2 Department of Bioengineering, University of Illinois Chicago, Chicago, IL 60607, USA; 3 Jesse Brown VA Medical Center, Chicago, IL 60612, USA; 4 Department of Microbiology and Immunology, University of Illinois Chicago, Chicago, IL 60612, USA; 5 UIC Cancer Center, University of Illinois Chicago, Chicago, IL 60612, USA

**Keywords:** archaeome, cobalamin, Inflammatory Bowel Disease (IBD), innate immunity, metabolite, microbiome, mycobiome, sulfate reduction, vitamin, vitamin D receptor, vitamin B12

## Abstract

A vitamin D receptor (VDR) deficiency leads to the dysbiosis of intestinal bacteria and is associated with various diseases, including cancer, infections, and inflammatory bowel disease. However, the impact of a VDR deficiency on fungi and archaea is unknown. We conditionally deleted the VDR in Paneth cells (VDR^ΔPC^), intestinal epithelial cells (VDR^ΔIEC^), or myeloid cells (VDR^ΔLyz^) in mice and collected feces for shotgun metagenomic sequencing and untargeted metabolomics. We found that fungi were significantly altered in each knockout (KO) group compared to the VDR^Loxp^ control. The VDR^ΔLyz^ mice had the most altered fungi species (three depleted and seven enriched), followed by the VDR^ΔPC^ mice (six depleted and two enriched), and the VDR^ΔIEC^ mice (one depleted and one enriched). The methanogen *Methanofollis liminatans* was enriched in the VDR^ΔPC^ and VDR^ΔLyz^ mice and two further archaeal species (*Thermococcus piezophilus* and *Sulfolobus acidocaldarius*) were enriched in the VDR^ΔLyz^ mice compared to the Loxp group. Significant correlations existed among altered fungi, archaea, bacteria, and viruses in the KO mice. Functional metagenomics showed changes in several biologic functions, including decreased sulfate reduction and increased biosynthesis of cobalamin (vitamin B12) in VDR^ΔLyz^ mice relative to VDR^Loxp^ mice. Fecal metabolites were analyzed to examine the involvement of sulfate reduction and other pathways. In conclusion, a VDR deficiency caused the formation of altered fungi and archaea in a tissue- and sex-dependent manner. These results provide a foundation about the impact of a host factor (e.g., VDR deficiency) on fungi and archaea. It opens the door for further studies to determine how mycobiome and cross-kingdom interactions in the microbiome community and metabolites contribute to the risk of certain diseases.

## 1. Introduction

The VDR is a highly conserved nuclear receptor [1,2,3]. While vitamin D has often been discussed for its role in the body’s mineral and skeletal homeostasis [2], the VDR is expressed in many cell types, including the small and large intestines of mammals [1,3,4]. There, it plays a critical role in innate and adaptive immunity, host–microbial interactions, proliferation, differentiation, and barrier function in a tissue-specific manner and related to infant and adult diseases [1,3,4,5].

Unsurprisingly, given this wide range of functions, the VDR and vitamin D deficiency have been linked with the pathogenesis of many diseases, including inflammatory bowel disease (IBD), colorectal cancer, and infections [1,3,6,7]. One of the mechanisms by which a vitamin D/VDR deficiency contributes to these diseases is by influencing host interactions with the microbiome [1,8,9]. Understanding the host–microbial interactions modulated by the VDR will provide new insights for preventing and treating these chronic diseases.

The intestinal bacteria, viruses, fungi, and archaea serve important functions in metabolism, immunity, and nervous system regulation [10,11,12]. Fungi constitute a minority of the inhabitants in the mammalian microbiome (in humans, around 0.1%) [13,14,15]. Despite their small numbers, fungi can have a major impact in the gut. The community of intestinal fungi (collectively called “the mycobiome”) has been shown to impact host immunity, metabolism, and vitamin availability [13,15,16]. Intestinal fungi also interact with other microbes, modulating their function and abundance [10,13,15,16]. Finally, the mycobiome can act as a repository for opportunistic pathogens that dangerously replicate and disseminate when given the opportunity, usually due to dysbiosis or compromised immune function [13,17].

To date, research on VDR deficiency’s impact on the microbiome has focused on bacteria and viruses [6,8,9,18]. It is unknown how a VDR deficiency impacts the mycobiome. Archaea are unicellular organisms in a separate domain from eukaryotes and bacteria that share several features of both and are often discovered under extreme environments [19,20]. Archaea form the least studied intestinal community, which is also known as “the archaeome”. The most abundant archaea in the mammalian intestine are methanogens, anaerobes that use the byproducts of bacterial fermentation to produce gaseous methane [20,21]. This links them to bacterial energy production—a notable interaction between these communities [19,22]. The archaeome is perhaps the least understood part of the microbiome but is an area of active research [19]. For example, alterations in archaea have been reported in populations with IBD [20,23], colorectal cancer (CRC) [24,25], and irritable bowel syndrome (IBS) [20]. However, the effect of a VDR deficiency on the archaeome has not been investigated.

In the current study, we hypothesized that a VDR deficiency alters the archaeome and mycobiome. To test our hypothesis, we used VDR conditional KO mouse models. These include the VDR KO in Paneth cells, intestinal epithelial cells, and myeloid cells, along with a VDR ^Loxp^ control group [8,26]. Fecal samples were collected from mice in each group for DNA isolation, followed by shotgun metagenomic sequencing. Differential abundance testing [27] revealed impacted fungi and archaea, which were linked to impacted bacteria and viruses via correlation analyses. We also tested sex subsets to investigate the sex-dependent actions of a VDR deficiency. Finally, functional metagenomic analyses and metabolite profiling provided more evidence for the impact of microbial perturbations vs. the VDR status in different tissues.

## 2. Materials and Methods

### 2.1. Animals

All the mice that were used in this study had a C57BL/6 genetic background. We conducted conditional deletions of the VDR in specific cell types of mice, including intestinal epithelial cells, Paneth cells, and myeloid cells [8]. In brief, to generate the VDR^ΔIEC^, VDR^ΔPC^, and VDR^ΔLyz^ mice, VDR^Loxp^ mice were crossed with villin-Cre, Defa6-Cre, and Lyz-Cre mice, respectively [8]. All the animals were housed in the Biologic Resources Laboratory (BRL) at the University of Illinois Chicago (UIC) and were utilized in accordance with the UIC Animal Care Committee (ACC) and the Office of Animal Care and Institutional Biosafety (OACIB) guidelines. The animal work was approved by the UIC Office of Animal Care (ACC15-231, ACC17-218, and ACC18-216). At the time of sample collection, all the animals were asymptomatic. In line with our power analysis, 10 mice (male and female, 6 to 8 weeks old) were assigned to each group for a total of 40 mice.

All the mice were housed in specific pathogen-free environments under a controlled condition of a 12 h light/12 h dark cycle at 20–22 °C and 45 ± 5% humidity, with free access to the same food and autoclaved water. All the materials that were utilized, including the cage, bedding, water bottles, and cage card holder, were autoclaved before housing the mice. The mice were housed with only mice of the same gender, and each cage had no more than 5 mice. All the mice that were subjected to our experiments were housed in groups before sample collection.

Further details about the breeding process can be found in a previous publication [8].

### 2.2. Shotgun Sequencing

In brief, fecal samples were collected from the colons of mice, sampled directly from the rectum, from each group with the following distribution: VDR^Loxp^ (male: *n* = 3 and female: *n* = 7), VDR^ΔIEC^ (male: *n* = 5 and female: *n* = 5), VDR^ΔPC^ (male: *n* = 5 and female: *n* = 5), and VDR^ΔLyz^ (male: *n* = 5 and female: *n* = 5). Age-matched mice were randomly selected for the control and experimental groups. DNA was extracted with DNeasy Power Fecal kits, homogenized using heat, and fragmented into short fragments (250–600 bp fragments) before sequencing on an Illumina HiSeq system.

Basic processing of the raw data were performed by the University of Illinois Chicago Core for Research Informatics (UICCRI). The data underwent quality control checks, filtering the reads, de-noising, and metagenomic assembly [28]. The resulting assemblies were filtered to exclude contigs shorter than 1000 nucleotides, and all remaining reads were classified with a centrifuge [29]. The taxonomic annotation of each contig was obtained by searching the NCBI GenBank non-redundant nucleotide database (as described in https://merenlab.org/2016/06/18/importing-taxonomy, accessed on 13 May 2019). Further details about the shotgun sequencing procedure can be found in our previous publication [8].

### 2.3. Metabolomics

Fecal samples were collected from the colons of chow-fed mice from a separate cohort spanning two KO groups with the following distribution: VDR^Loxp^ (male: *n* = 10 and female: *n* = 6), VDR^ΔIEC^ (male: *n* = 9 and female: *n* = 8), and VDR^ΔLyz^ (male: *n* = 5 and female: *n* = 5). The samples were prepared using the MicroLab STAR^®^ system, preceded by the addition of several recovery standards for QC purposes. To separate bound small molecules, this was followed by protein precipitation with methanol and shaking for 2 min before centrifugation. TurboVap (Zymark, Hopkinton, MA, USA) was used to remove the organic solvent before overnight storage in nitrogen. Four fractions of this extract were sent for mass spectrometry analysis—one via the reverse phase (RP)/UPLC-MS/MS method with negative ion mode electrospray ionization (ESI), one via HILIC/UPLC-MS/MS with negative ion mode ESI, and two via separate RP/UPLC-MS/MS with positive ESI. Further details can be found in our previous publication [30].

### 2.4. Statistical Analysis

Two-sided tests of significance were performed in all the analyses. For differential abundance, correlation, and metagenomic functional analyses, the statistical significance was evaluated using *q*-values that corrected for the false discovery rate (FDR). Unless stated otherwise, a significance value of *p* ≤ 0.05 was considered statistically significant. The R Foundation for Statistical Computing Platform (version 4.2.2, 31 October 2022, Vienna, Austria) was used for the statistical analyses and plotting.

### 2.5. Microbiome Data Analysis

#### 2.5.1. Alpha Diversity

Alpha diversity measures the total diversity within microbial communities/samples [31]. The Shannon diversity metric, which takes into account both species richness and evenness [32], was used to quantify the diversity of fungal species. The Shapiro–Wilk test was used to assess the distribution of alpha diversity measures, and it was found that they were normally distributed, which confirmed the appropriateness of using general linear models in this analysis. However, for the purpose of comparisons, Shannon diversity was analyzed using both general linear models and Mann–Whitney rank-sum tests controlling for sex.

#### 2.5.2. Beta Diversity

Beta diversity measures the difference in diversity between two communities by evaluating how much one distribution of microbial taxa differs from another [33]. The Bray–Curtis dissimilarity metric was used to quantify beta diversities as follows:

$${BC}_{ij}=1-\frac{2C_{ij}}{S_{i}+S_{j}}$$

where *C_ij_*—the sum of lesser counts of each species found in both groups; *S_i_*—total count for all species in group *i* and *S_j_*—total count for all species in group *j*.

In simple terms, this metric reports one minus the ratio of matching counts found in both samples across all species to the total number of counts found in both samples. A larger Bray–Curtis value indicates that two distributions are further apart [33].

First, an analysis of similarities (ANOSIM) was applied to compare dissimilarities between and within genotypes at the species level. Since the results showed that there were statistically significant differences (*p* < 0.05), the same non-parametric test was performed on the data of genotype and sex. Further ANOSIM tests were applied for each sex subset of all control–experimental pairs and between sex subsets of each experimental group. Finally, a non-parametric PERMANOVA was used to evaluate the impact of sex on beta diversity and the interaction term between sex and genotype [34].

#### 2.5.3. Differential Abundance

Differential abundance was performed using the Analysis of Compositions of Microbiomes with Bias Correction 2 (ANCOM-BC2) [27]. This is a method that is used for differential abundance analysis that corrects biases caused by differences in sampling fractions, and it was selected as a conservative approach based on the review by Nearing et al. [35]. Default parameters were used, and the program was run multiple times for genotype and genotype plus sex interactions. All archaea and fungi that were statistically differentially abundant (*q*-value ≤ 0.1) in comparing an experimental group to their control with a magnitude of log2 fold change ≥ 0.1 were compiled and reported.

#### 2.5.4. Correlation Analysis

Correlation analysis was performed on the microbial taxa that were found to be differentially abundant in each experimental group in this study (see Section 2.5.3) and our previous publication [8]. Before running this analysis, Shapiro–Wilk tests were performed to determine the normality of the data of the microbial taxa. Given that the differentially abundant microbial taxa were not normally distributed via these tests, we performed a Spearman correlation with the Benjamin–Hochberg correction using the Misty R package [36]. We did not report the correlations between pairs of differentially abundant taxa that were not significantly correlated with each other. A similar analysis was performed on the results from the sex subset ANCOM-BC2 analysis, although it revealed few new correlation patterns (Supplementary Figures S1 and S2).

#### 2.5.5. Fungi Annotation

Shotgun sequencing methods often report fungi that are incapable of colonizing the gut [37,38,39]. These findings, often caused by food, contamination [17], or annotation errors [40], can distract from the fungi that are the most biologically relevant. To combat this, we implemented a literature search to annotate the fungal taxa into two groups—those with a low likelihood of being biologically relevant in the mammalian gut and those with a high likelihood of being biologically relevant. This step did not filter or remove any taxa.

First, we utilized the curated literature review by Suhr et al. [37], which identified the fungi confirmed by multiple sources to be inhabitants of the mammalian gut, to rule in relevant organisms as having a high likelihood. Next, we labeled as less likely those fungi from groups that are incapable of surviving in the gut, as described by Suhr et al. and Lavrinienko et al. [37,38,39]. These include edible macrofungi [37,38], lichen fungi [38], plant pathogens and endophytes [37,38,39], and wood-decaying fungi [37,39]. Soil fungi were deemed high likelihood, as they have been reported as opportunistic pathogens, even if the gut is not their preferred niche [37,39]. Finally, entomopathogenic fungi, which specifically infect insects [41], were flagged as less likely.

The fungi were sorted into these categories for classification using the FUNGuild functional database [42]. This database is sparse and, when a taxa was missing, it was manually categorized based on the literature-established properties of that taxa. For edible macrofungi, the genus *Morchella* [43], the genus *Amanita* [44], *Hypsizygus marmoreus* [45], and *Coprinus comatus* were uncovered [46]. For entomopathogens, the genus *Hirsutella* [47], *Tetrapisispora blattae* [48], and *Metarhizium acridum* were identified [49]. For plant endophytes, *Rhizophagus custos* [50] and *Sarocladium zeae* were uncovered [51]. For plant commensals, the genus *Starmerella* [52] and *Candida magnoliae* were uncovered [53]. For plant pathogens, the genus *Puccinia* [54], *Heterobasidion irregulare* [55], and *Colletotrichum gloeosporioides* were identified [56]. For wood-decaying fungi, the genus *Coprinopsis* [57] and *Phlebia tremellosa* were identified [58]. For lichen, *Sarcogyne privigna* was uncovered [59].

Differential abundance results with these annotations can be found in Supplementary Figure S3.

#### 2.5.6. Functional Analysis

Functional analysis predicts differences in potential functionality of the overall (archaeal, fungal, bacterial, and viral) microbiomes of control animals versus those of the experimental groups. This method examines all the sequenced genes in each community and their functions (see Section 2.2), regardless of their microbe of origin, and uses this information to build functional profiles for each community at the individual gene, module, and pathway levels [60]. Functional analysis was conducted to compare the statistically significant differences (*q* ≤ 0.05) between each of the experimental profiles and their controls.

#### 2.5.7. Metabolomics Analysis

The metabolomics data were analyzed by applying the Welsh’s two-sample *t*-test on the log-transformed data for the VDR^ΔLyz^ vs. VDR^Loxp^ and VDR^ΔLyz^ vs. VDR^ΔIEC^ comparisons and through applying an ANOVA test on the log-transformed data for the VDR^ΔIEC^ vs. VDR^Loxp^ comparison [8]. Effect sizes were evaluated via log2 fold changes compared to the reference group.

## 3. Results

### 3.1. Conditional VDR KO Alters Fungal Beta Diversity

**Figure 1:**
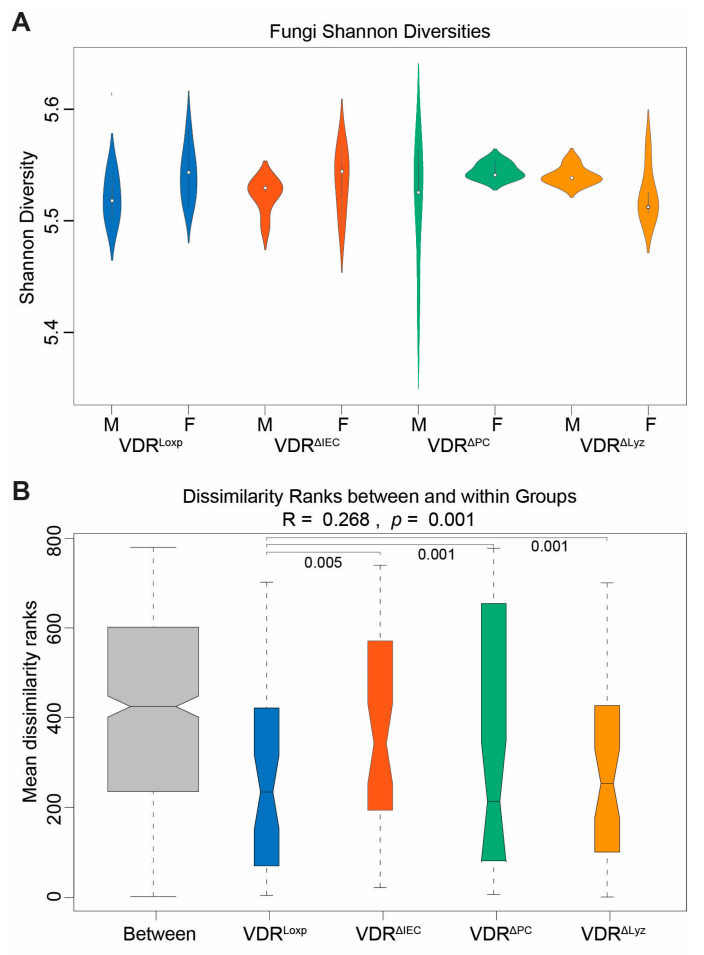
Species-level alpha and beta diversity via different tissue deletions of the VDR. (**A**) Distribution of Shannon diversity metrics at the species level, divided by sex, for fungi. Color indicates genotype. Subsequent ANOSIM analysis on sex subgroups revealed no statistically significant comparisons (*p* > 0.05). (**B**) Within-group versus between-group Bray–Curtis dissimilarity metrics for mice of both sexes. Color indicates genotype. Subsequent ANOSIM analysis found all control-knockout pairs to be significantly different. These results, VDR^ΔIEC^ vs. VDR^Loxp^ (*p* = 0.005), VDR^ΔPC^ vs. VDR^Loxp^ (*p* = 0.001), and VDR^ΔLyz^ vs. VDR^Loxp^ (*p* = 0.001), are indicated on the boxplot.

No significant differences in fungal Shannon diversity were found between the control VDR^Loxp^ group and any of the VDR^ΔIEC^, VDR^ΔPC^, and VDR^ΔLyz^ groups (Figure 1A). A general linear model that controlled for sex was then applied to compare the Shannon diversities of each experimental group with the control group. No significant difference was found in a gender-specific manner.

Regarding the beta diversities, all the knockout groups encompassed distributions of the fungal species that were significantly different from the distributions of the VDR^Loxp^ control group (*p* < 0.05) (Figure 1B).

### 3.2. Differentially Abundant Fungi Vary by VDR KO in Different Tissues

Having shown that a VDR deletion changes the overall fungal communities, we next investigated which specific members of those communities were impacted by searching for microbes that were differentially abundant between the KO and control groups. This was carried out using the Analysis of Compositions of Microbiomes with Bias Correction 2, a conservative differential abundant toolkit that controls for biases due to sampling [27]. The findings that were uncovered using the ANCOM-BC2 toolkit were followed by the application of significance (*q* ≤ 0.1) and fold change thresholds (│log_2_(Fold change)│ ≥ 0.1) to narrow down the relevant taxa.

**Figure 2:**
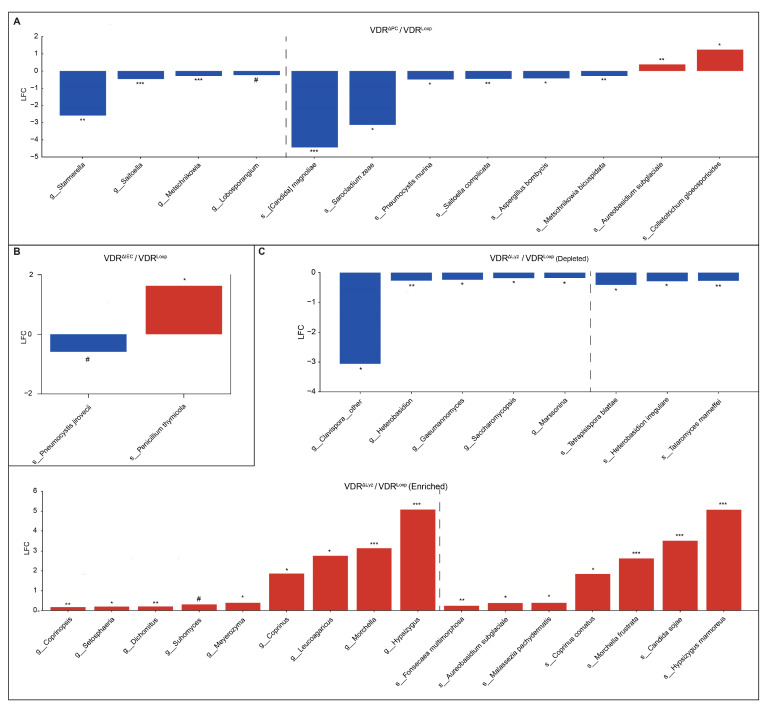
Differential fungi in the KO mice. Fungal taxa that are significantly (*q*, # ≤ 0.1, * ≤ 0.05, ** ≤ 0.01, and *** ≤ 0.001) differentially abundant in VDR KO mice relative to the control group, not sub-dividing by sex. “s_” indicates that the taxa is a species and “g_” indicates that it is a genus—the only two taxonomic levels that were studied. LFC indicates log2 fold change, and the color indicates the direction of the shift relative to the control (blue is decreased, and red is increased). (**A**) VDR^ΔPC^, (**B**) VDR^ΔIEC^, and (**C**) VDR^ΔLyz^.

Each VDR KO group had distinct fungi relative to the control group over a wide range of log2 fold changes (from −4.5 to 5.0) (Figure 2). The VDR^ΔPC^ group exhibited a decrease in ten such taxa (including the genera *Starmerella*, *Saitoella*, *Metschnikowia*, and *Lobosporangium*, along with the species *Candida magnoliae*, *Sarocladium zeae*, *Pneumocystis murina*, *Saitoella complicata*, *Aspergillus bombycis*, and *Metschnikowia bicuspidata*) and an increase in two taxa (the species *Aureobasidium subglaciale* and *Colletotrichum gleosporioides*). The VDR^ΔIEC^ group showed a decrease in a single species (*Pneumocystis jirovecii*) and an increase in a single species (*Penicillum thymicola*). Finally, the VDR^ΔLyz^ group displayed eight taxa that were depleted (the genera *Heterobasidion, Gaeumannomyces, Saccharomycopsis,* and *Marssonina*, along with the species *Tetrapisispora blattae, Heterobasidion irregulare, Talormyces marneffei,* and an unidentified *Clavispora* species) relative to the VDR^Loxp^ group and sixteen that were relatively enriched (the genera *Coprinopsis, Setosphaeria, Dichomitus, Suhomyces, Meyerozyma, Coprinus, Leucoagaricus, Morchella,* and *Hypsizygus*, along with the species *Fonsecaea multimorphosa*, *Aureobasidium subglaciale*, *Malassezia pachydermatis*, *Coprinus comatus*, *Morchella furstrata*, *Canddia sojae*, and *Hypsizygus mammoreus*).

### 3.3. Significant Fungal Correlations Exist with Archaea, Bacteria, and Viruses

**Figure 3:**
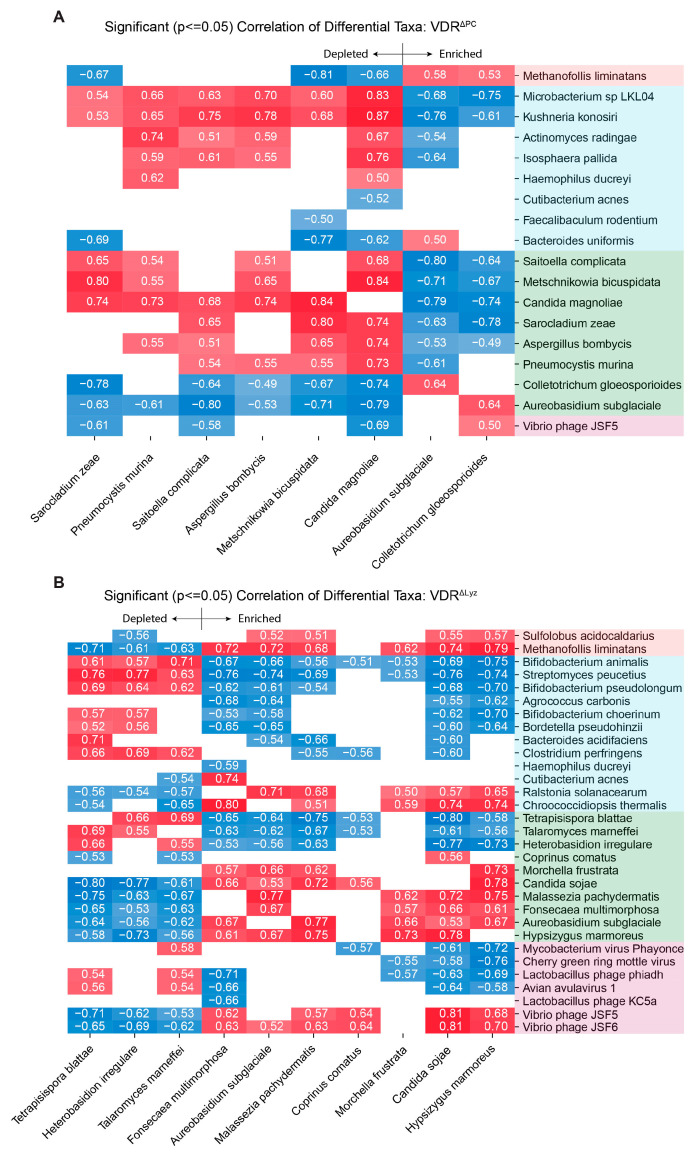
Significant (*p* ≤ 0.05) correlations between significantly differentially abundant (*q* ≤ 0.1) species of fungi and significantly differentially abundant (*q* ≤ 0.1) bacteria, archaea, and viruses. The cell color indicates the magnitude of the correlation (blue = negative; red = positive), while a white space indicates no significant correlation. The colors of the row names indicate the following: peach for archaea, blue for bacteria, green for eukaryotes, and purple for viruses. (**A**) VDR^ΔPC^. (**B**) VDR^ΔLyz^.

We performed correlation analyses to study how knockout-induced changes in fungi may relate to the changes in archaeal, bacterial, and viral populations. Those pairs of differentially abundant microbial taxa (*q* ≤ 0.1) that have significant correlations (*p* ≤ 0.05) are displayed (Figure 3). Decreasing correlation patterns exhibited positive correlations with the fungi that were less abundant in the knockout mice and negative correlations with the fungi that were more abundant. Increasing correlation patterns displayed positive correlations with the fungi that were more abundant in the KO mice and negative correlations with the fungi that were less abundant.

Within the VDR^ΔPC^ group, multiple bacterial species (*Microbacterium* sp LKL104, *Isosphaera pallida*, *Kushneria konosiri*, and *Actinomyces radingae*) and several fungal species (*Saitoella complicate*, *Metschnikowia bicuspidata*, *Candida magnolia*, *Sarocladium zeae*, *Aspergillus bombycis*, and *Pneumocystis murina*) follow a decreasing correlation pattern (Figure 3A). One archaeal species (*Methanofollis liminatans*), one bacterial species (*Bacteroides uniformis*), two fungal species (*Colletotrichum gloeosporioides* and *Aureobasidium subglaciale*), and one virus (Vibrio phage JSF5) followed an increasing correlation pattern. In contrast, there were no significant correlation pairs in the VDR^ΔIEC^ group. Within the VDR^ΔLyz^ group, however, many bacterial species (*Bacteroides acidifaciens*, *Bifidobacterium animalis*, *Bifidobacterium choerinum*, *Bifidobacterium pseudolongum*, *Haemophilus ducreyi*, *Clostriduium perfringens*, *Bordetella pseudohinzii*, *Agrococcus carbonis*, and *Streptomyces peucetius*), three fungal species (*Tetrapisispora blattae*, *Talaromyces marneffei*, and *Heterobasidion irregulare*), and several viruses (*Lactobacillus phage KC5a, Lactobacillus phage phiadh, Mycobacterium virus phayonce, Avian avulvavirus 1*, and *Cherry green ring mottle virus*) followed a decreasing correlation pattern. Two archaeal species (*Methanofollis liminatans* and *Sulfolobus acidocaldarius*), three bacterial species (*Cutibacterium acnes*, *Chroococcidiopsis thermalis*, and *Ralstonia solanacaerum*), seven fungal species (*Coprinus comatus*, *Morchella frustrata*, *Candida sojae*, *Malassezia pachydermatis*, *Fonsecea multimorphosa*, *Aurobasidium subglaciale*, and *Hypsizygus marmoreus*), and two vibrio phages (Vibrio phage JSF5 and JSF6) followed an increasing correlation pattern.

### 3.4. Sex Differences of Fungal Dysbiosis in the VDR KO Mice

The fungal data were split by sex, and the beta diversity of the fungal data was further tested for gender differences (Supplementary Figure S4). A subsequent PERMANOVA test [34] revealed a significant (*p* = 0.002) sex difference between male and female VDR^ΔLyz^ mice. ANOSIM statistical testing [34] on the sex subsets showed that, while all female experimental groups had marginally significantly (*p* < 0.1) different fungal distributions from the female control group, only the male VDR^ΔLyz^ group had a marginally significant (*p* = 0.081) different distribution from the male VDR^ΔLyz^ group (Supplementary Figure S4). What is more, ANOSIM testing between sex groups within each group revealed significant differences (*p* < 0.05) in the distribution of fungi within each KO group but not within the control group.

**Figure 4:**
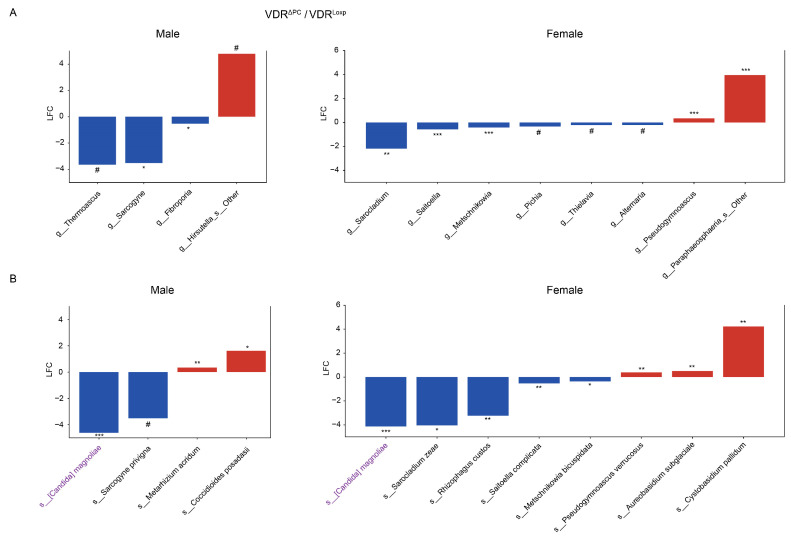
Differential fungi in sex subsets of the VDR^ΔPC^ group. Fungal taxa that are significantly (*q* # ≤ 0.1, * ≤ 0.05, ** ≤ 0.01, and *** ≤ 0.001) differentially abundant in the VDR knockout mice relative to the control mice via sex subset. Figure (**A**) shows the genera and figure (**B**) shows the species—the only two taxonomic levels that were observed. LFC indicates the log2 fold change, and the color indicates the direction of the shift relative to the control (blue is decreased, and red is increased). Purple font indicates that a taxa was found to be differentially abundant in both the male and female groups.

**Figure 5:**
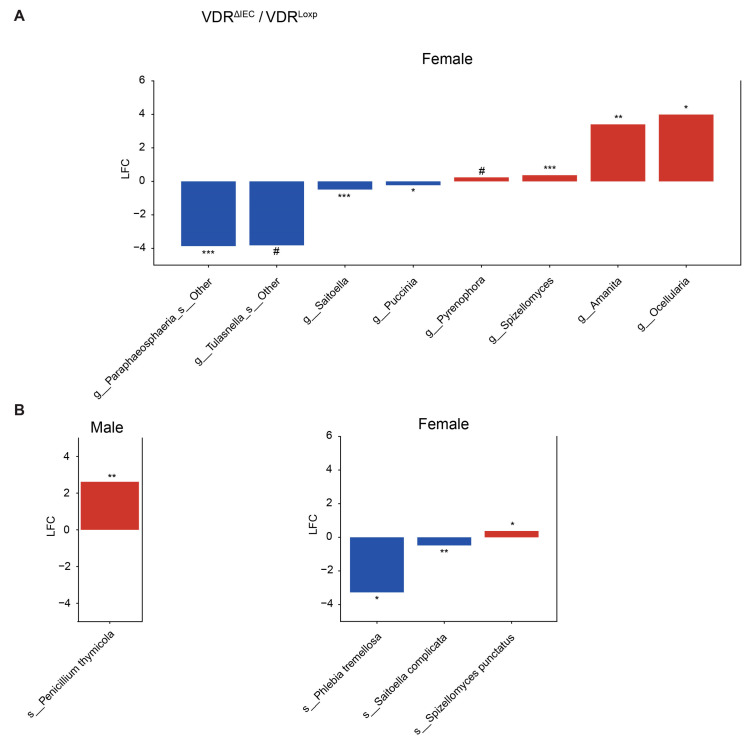
Differential fungi in sex subsets of the VDR^ΔIEC^ group. Fungal taxa that are significantly (*q* # ≤ 0.1, * ≤ 0.05, ** ≤ 0.01, and *** ≤ 0.001) differentially abundant in the VDR knockout mice relative to the control mice via sex subset. Figure (**A**) shows the genera and figure (**B**) shows the species—the only two taxonomic levels that were observed. LFC indicates the log2 fold change, and the color indicates the direction of the shift relative to the control (blue is decreased, and red is increased). Purple font indicates that a taxa was found to be differentially abundant in both the male and female groups.

**Figure 6:**
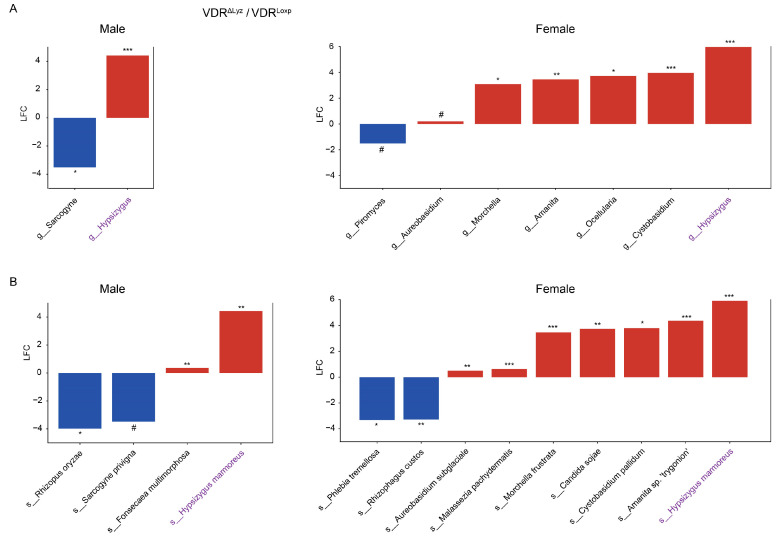
Differential fungi in sex subsets of the VDR^ΔLyz^ group. Fungal taxa that are significantly (*q* # ≤ 0.1, * ≤ 0.05, ** ≤ 0.01, and *** ≤ 0.001) differentially abundant in the VDR knockout mice relative to the control mice via sex subset. Figure (**A**) shows the genera and figure (**B**) shows the species—the only two taxonomic levels that were observed. LFC indicates the log2 fold change, and the color indicates the direction of the shift relative to the control (blue is decreased, and red is increased). Purple font indicates that a taxa was found to be differentially abundant in both the male and female groups.

The use of ANCOM-BC2 [27] on the sex subsets revealed distinct differential fungi in each subset (Figure 4, Figure 5 and Figure 6). None of the twelve differentially abundant genera (four in males and eight in females) in the subsets of the VDR^ΔPC^ mice were found in both the male and female subsets (Figure 4A), and only *Candida magnoliae* of the eleven differentially abundant species (three in only male, seven in only female, and one in both) was found in both subsets (Figure 4B).

The sex subsets of the VDR^ΔIEC^ mice shared no differentially abundant taxa from the eight genera (eight in males) (Figure 5A) and four species (one in males and three in females) (Figure 5B) that were found to be significantly differentially abundant relative to the control mice.

In the VDR^ΔLyz^ group, the genus *Hypsizugus* was the only one of the eight differentially abundant genera (one in only males, seven in only females, and one in both) that was found in both groups (Figure 6A), and its member *Hypsizygus mamoreus* was the only one of the twelve differentially abundant species (three in only males, eight in only females, and one in both) that was found in both sex subsets (Figure 6B).

### 3.5. VDR Knockouts Alter Archaeal Alpha and Beta Diversities

**Figure 7:**
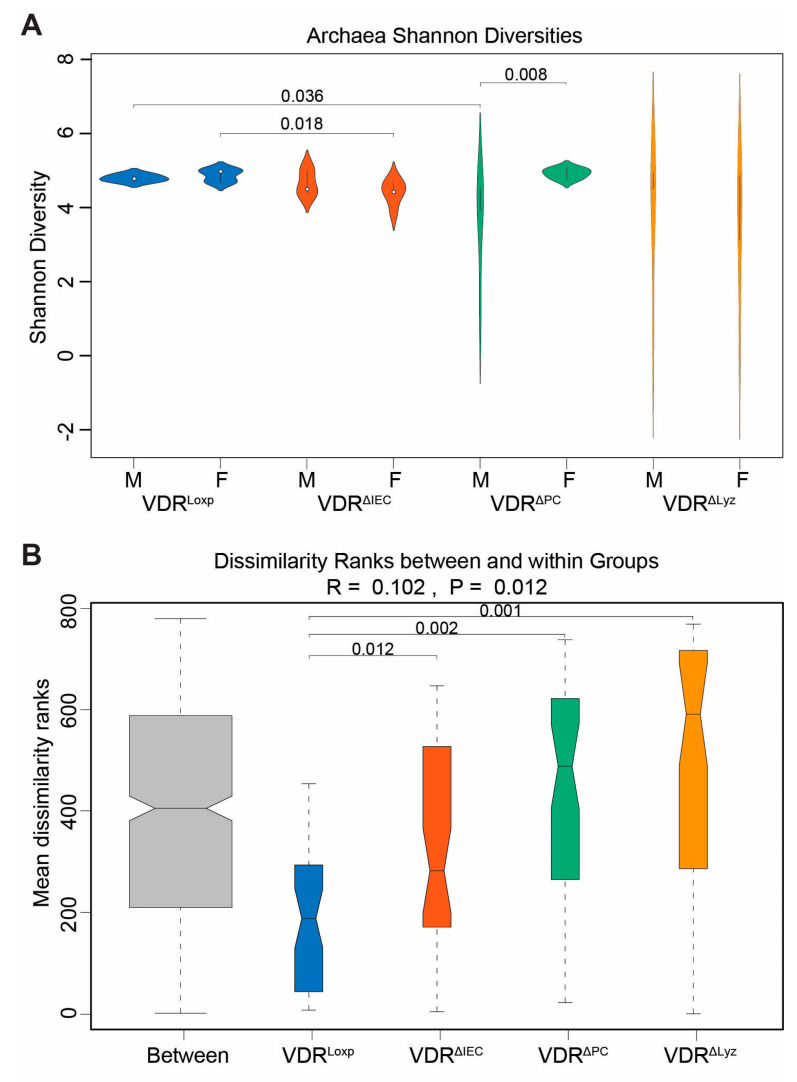
Species-level alpha and beta diversity in the VDR KO mice compared to the VDR^Loxp^ control group. (**A**) Shannon diversity by sample. Presented are the *p*-values of the subsequent ANOSIM analysis on the sex subgroups that were statistically significant (*p* ≤ 0.05) or marginally significant (*p* ≤ 0.1). (**B**) Within-group versus between-group Bray–Curtis dissimilarity metrics for mice of both sexes. Color indicates genotype. A subsequent ANOSIM analysis found all the control–knockout pairs to be significantly different. These results, VDR^ΔIEC^ vs. VDR^Loxp^ (*p* = 0.012), VDR^ΔPC^ vs. VDR^Loxp^ (*p* = 0.002), and VDR^ΔLyz^ vs. VDR^Loxp^ (*p* = 0.001), are indicated on the boxplot.

In Figure 7A, the alpha diversity of archaea was analyzed. There was a statistically significant (*p* < 0.05) drop in the alpha diversity of archaeal species in male VDR^ΔPC^ mice relative to male VDR^Loxp^ mice. There was also a similarly significant (*p* < 0.05) drop in the alpha diversity of archaeal species in female VDR^ΔIEC^ mice relative to female VDR^Loxp^ mice. In the beta diversity analysis, all the knockout groups were found to have species distributions that were significantly different from the distribution of the VDR^Loxp^ control group (*p* < 0.05) (Figure 7B).

### 3.6. Common Set of Abundant Archaea in the VDR^ΔPC^ and VDR^ΔLyz^ Groups

**Figure 8:**
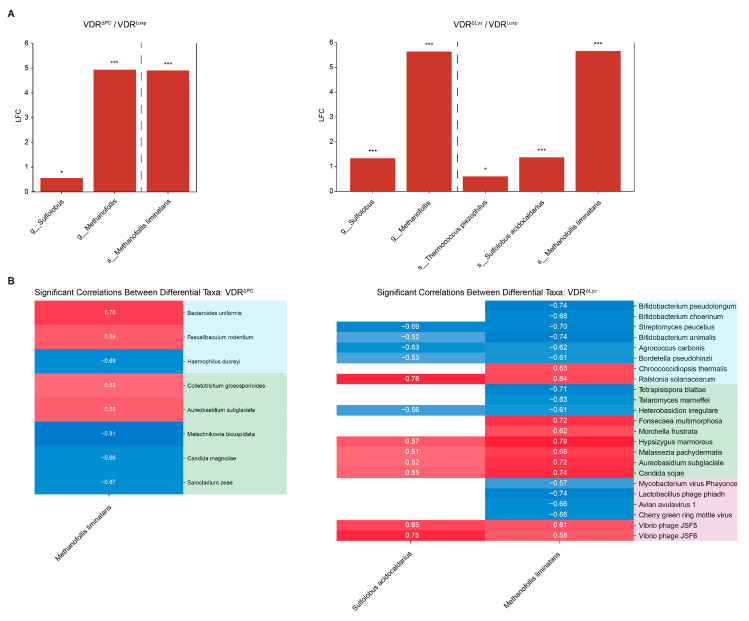
Differential archaea and their correlation maps. (**A**) Archaeal taxa that are significantly (*q* ≤ 0.1, * ≤ 0.05, and *** ≤ 0.001) differentially abundant in the VDR KO mice relative to the control group, not splitting by sex. “s_” indicates that the taxa is a species and “g_” indicates it is a genus—the only two taxonomic levels that were used as inputs. LFC indicates the log2 fold change, and the color indicates the direction of the shift relative to the control (blue is decreased, and red is increased). (**B**) Significant (*p* ≤ 0.05) correlations between significantly (*q* ≤ 0.1) differentially abundant species of archaea and significantly (*q* ≤ 0.1) differentially abundant bacteria, fungi, and viruses. The color indicates the magnitude of the correlation (blue = negative; red = positive), while a white space indicates no significant correlation. The colors of the row names indicate the following: peach for archaea, blue for bacteria, green for eukaryotes, and purple for viruses.

The differential abundance of archaeal taxa between the control and knockout groups was performed as in the fungal analysis (see Section 3.2) (Figure 8A). Three taxa (the genera *Sulfolobus* and *Methanofollis*, along with the species *Methanofollis limitans*) were more abundant in both the VDR^ΔPC^ and VDR^ΔLyz^ groups compared to the control mice. Two other archaeal species (*Thermococcus piezophilus* and *Sulfolobus acidocaldarius*) were only increasingly abundant in the VDR^ΔLyz^ group. There were no differential taxa in the VDR^ΔIEC^ group compared to the VDR^Loxp^ group.

### 3.7. Signficant Archaeal Correlations Exist with Bacteria, Fungi, and Viruses

In Figure 8B, correlation analyses were performed using all the differential archaeal species. Within the VDR^ΔPC^ group, two bacterial species (*Bacteroides uniformis* and *Faecalibaculum rodentium*) and two fungal species (*Colletrotrichum gloeosporioides* and *Aureobasidium subglaciale*) were positively correlated with *Methanofolis liminatans*, which is abundant relative to the control group. The bacteria *Haemophilus ducreyi* and three fungal species (*Metschnikowia bicuspidata*, *Candida magnoliae*, and *Sarocladium zeae*) were negatively correlated with *Methanofolis liminatans*. Within the VDR^ΔPC^ group, many bacteria (*Bifidobacterium animalis*, *Bifidobacterium choerinum*, *Bifidobacterium pseudolongum*, *Bordetella pseudohinzii*, *Agrococcus carbonis*, and *Streptomyces peucetius*), three fungal species (*Tetrapisispora blattae*, *Talaromyces marneffei*, and *Heterobasidion irregulare*), and several viruses (*Lactobacillus phage phiadh, Mycobacterium virus phayonce, Avian avulavirus 1,* and *cherry green ring mottle virus*) were negatively correlated with relatively abundant archaea. Additionally, several bacteria (*Chroococcidiopsis thermalis* and *Ralstonia solanacearum*), six fungi (*Fonsecaea multimorphosa*, *Morchella frustrata*, *Hypsizygus marmoreus*, *Malassezia pachydermatis*, *Aureobasidium subglaciale*, and *Candida sojae*), and two vibrio phages (*Vibrio* phages *JSF5* and *JSF6*) were positively correlated with the archaea that were abundant in the KO group relative to the control group.

### 3.8. The Archaeal Dysbiosis Caused by VDR Deletions Is Sex Dependent

The drop in total diversity of archaeal species was specific to certain sex subsets (male VDR^ΔPC^ and female VDR^ΔIEC^), and the male and female VDR^ΔPC^ mice have significantly (*p* < 0.05) different total diversities (Figure 7A). Next, as in the fungal analysis, beta diversity was calculated on the sex subsets (Supplementary Figure S5). The subsequent PERMANOVA test [34] revealed a non-significant (*p* > 0.05) sex–genotype interaction term. However, ANOSIM statistical testing on the sex subsets showed that, while all the female experimental groups had marginally significantly (*p* < 0.1) different archaeal distributions from the female control group, none of the male sub-groups had a marginally significantly (*p* < 0.1) different distribution (Supplementary Figure S5). Additional ANOSIM testing between sex groups within each group revealed significant differences (*p* = 0.01) between the archaeal distributions of the male and female VDR^ΔPC^ mice.

**Figure 9:**
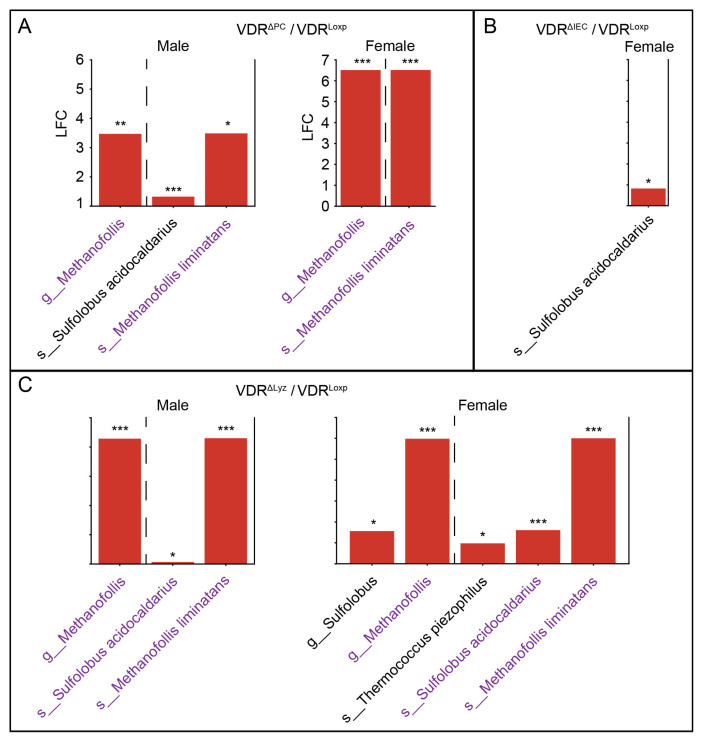
Differential archaea in sex subsets. Archaeal taxa that are significantly (*q* ≤ 0.1, * ≤ 0.05, ** ≤ 0.01, and *** ≤ 0.001) differentially abundant in VDR knockout mice. (**A**) VDR^ΔPC^, (**B**) VDR^ΔIEC^, and (**C**) VDR^ΔLyz^ in male and female mice. “s_” indicates that the taxa is a species and “g_” indicates that it is a genus—the only two taxonomic levels that were observed. LFC indicates the log2 fold change, and the color indicates the direction of the shift relative to the control (blue is decreased, and red is increased).

The use of ANCOM-BC2 on the sex subsets produced largely overlapping taxa (Figure 9). The two *Methanofollis* taxa (the genus *Methanofollis* and the species *Methanofollis liminatans*) were enriched in both sex subsets of the VDR^ΔPC^ (Figure 9A) and VDR^ΔLyz^ (Figure 9C) groups relative to their matched control groups. One species (*Sulfolobus acidocaidarius*) was relatively enriched in the male subset of the VDR^ΔPC^ group but not the female subset (Figure 9A). In contrast, this species was abundant relative to Loxp in the female subset of the VDR^ΔIEC^ group but not the male subset (Figure 9B). *Sulfolobus acidocaidarius* was also enriched relative to Loxp in both sex subsets of the VDR^ΔLyz^ group, but its genus (*Sulfolobus*) was only relatively enriched in the female subset (Figure 9C). Finally, *Thermococcus piezophillus* was enriched relative to the control mice in the female but not the male VDR^ΔIEC^ subdivisions (Figure 9C).

### 3.9. Functional Metagenomics

**Figure 10:**
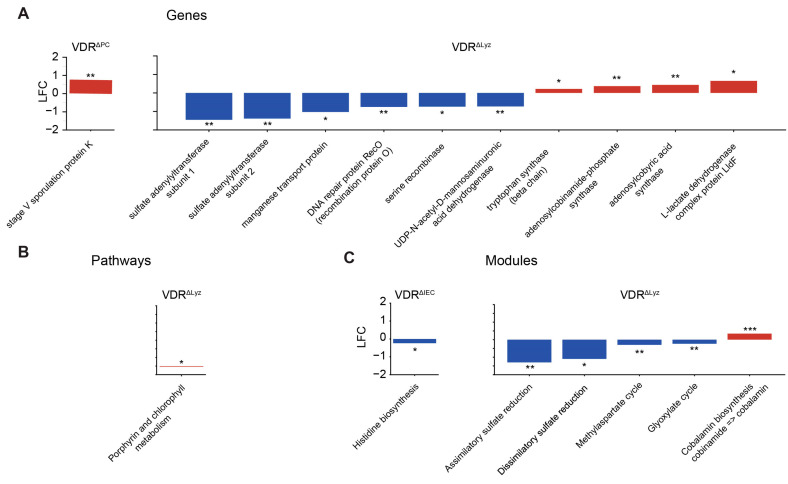
Predictive functional analysis using metagenomics. (**A**) Genes, (**B**) pathways, and (**C**) modules that are significantly (*q* ≤ 0.1, * ≤ 0.05, ** ≤ 0.01, and *** ≤ 0.001) increased in the KO vs. control mice. The magnitude of this difference is given as the log2 fold change (LFC), and the color indicates the direction of the shift relative to the control.

Functional metagenomics was performed to investigate differences in the predictive functional capacities of the microbiomes in the control and KO mice. All genes found to be significantly different between the control group and a KO group except one (stage five sporulation protein k, increased in the VDR^ΔIEC^ mice relative to VDR^Loxp^ mice) originate from the VDR^ΔLyz^ to VDR^Loxp^ comparison (Figure 10A). Most of these were reduced in the KO group (sulfate adenyltransferase subunits 1 and 2, manganese transport protein, DNA repair protein recombination protein O, serine recombinase, and UDP-N-acetyl-D-mannosaminuronic acid dehydrogenase) relative to the control group; however, four were increased (*tryptophan synthase* (beta chain), adenosylcobinamide-phosphate synthase, adenylcobyric acid synthase, and the L-lactate dehydrogenase complex protein Lldf). Only a single pathway, porphyrin and chlorophyl metabolism, was found to deviate between any of the KO groups and the control group (Figure 10B). This pathway was significantly increased in the VDR^ΔLyz^ mice. Three modules were predicted to differ between the VDR^ΔLyz^ and VDR^Loxp^ groups (Figure 10C). These included a predicted decrease in two sulfate reduction modules (assimilatory sulfate reduction and dissimilatory sulfate reduction) relative to the control mice and a predicted increase in the vitamin B12 (cobalamin) biosynthesis module. The genomes of four microbes that were differentially abundant in the VDR^ΔLyz^ group contained genes relating to these modules (Table 1).

### 3.10. Metabolomic Signiture Related to VDR KO in Myeloid Cells

**Figure 11:**
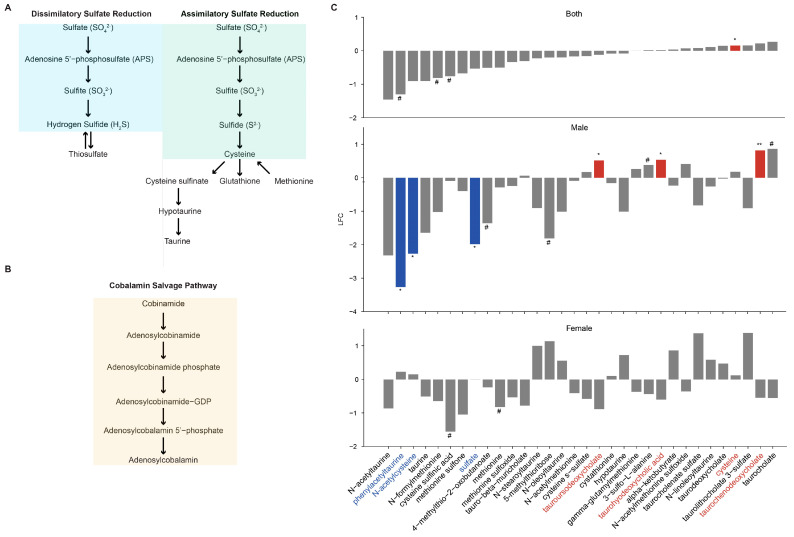
Metabolites related to sulfate reduction. (**A**) The primary metabolites involved in dissimilatory and sulfate reduction, along with key downstream molecules [62,63]. (**B**) The primary metabolites involved in the cobalamin salvage pathway [64]. (**C**) The difference in the abundances of metabolites from the above pathways detected in the VDR^ΔLyz^ mice relative to the VDR^Loxp^ mice. LFC indicates the Log2 fold change. Gray indicates a lack of statistical significance (*p* ≤ 0.05), while the presence of a significant difference is given by non-gray color and * annotation. A # indicates marginal significance (*p* ≤ 0.1).

To investigate/validate the functional impacts of these predicted changes, we analyzed the fecal metabolomes of a separate cohort of VDR^ΔLyz^, VDR^ΔIEC^, and VDR^Loxp^ mice. After analyzing the overall changes in these metabolites, we focused on the metabolites relating to sulfate reduction (Figure 11A) and cobalamin salvage (Figure 11B) pathways within the VDR^ΔLyz^ vs. VDR^Loxp^ comparison. The VDR^ΔLyz^ mice had a significant (*p* < 0.05) increase in cysteine, the immediate product of assimilatory sulfate reduction (Figure 11C), relative to the VDR^Loxp^ mice. Furthermore, the male subgroup of the VDR^ΔLyz^ mice had a significantly lower abundance of sulfate itself, the primary input of both assimilatory and dissimilatory sulfate reduction, relative to the control mice.

## 4. Discussion

In the current study, we have demonstrated that fungi and archaea were significantly altered in each VDR KO group compared to the VDR^Loxp^ control. The VDR^ΔLyz^ mice had the most altered fungal species (three depleted and seven enriched), the VDR^ΔPC^ mice the second most (six depleted and two enriched), and the VDR^ΔIEC^ mice had the fewest (one depleted and one enriched). The methanogen *Methanofollis limitans* was enriched in the VDR^ΔPC^ and VDR^ΔLyz^ mice, and two further archaeal species (*Thermococcus piezophilus* and *Sulfolobus acidocaldarius*) were enriched in the VDR^ΔPC^ mice compared to the VDR^Loxp^ group. Significant correlations existed among altered fungi, archaea, bacteria, and viruses in the KO mice. Functional metagenomics showed changes in several biologic functions. These results provide a foundation about the impact of a VDR deficiency, as a host factor, on fungi and archaea and cross-kingdom interactions in the microbiome community.

Disruption to the normal microbiome leads to an imbalance and increases susceptibility to further perturbations. This is why dysbiosis is often associated with pathology, for instance, drops in archaeal diversity like those seen here have been reported in obesity [65] and amyotrophic lateral sclerosis (ALS) [66], suggesting that this trend can either be a biomarker or a driver of an illness. Fungal dysbiosis is also common in disease states [67], likely due to the important impact that the mycobiome has on host immunity [68]. Different types of fungal dysbiosis have been linked to colorectal cancer (CRC) [69] and Crohn’s disease [70]. In most pathologic states (infection, inflammatory disease, etc.), a VDR deficiency is one of many factors contributing to pathogenesis [1,9,71]. Our model targets one specific gene in a tissue-specific manner and provides the evidence for host factors in shaping the microbiome and modulating the risk of diseases.

The VDR regulation of fungi and archaea occurs in a tissue-specific manner, evidenced by the differential archaea and fungi across the KO groups. The VDR^ΔLyz^ mice harbored multiple opportunistic intestinal pathogens in increased abundance. The increase in three types of Candida (the genera *Myerozyma* and *Suhomyces*, along with the species *Candida sojae*) in these mice relative to the control mice is also noteworthy, as the relative abundance of Candida has been reported in inflammatory intestinal illnesses [72] and specifically Crohn’s disease [65]. Additionally, the relative abundance of *M. pachydermatis* in the myeloid knockouts may be significant, as human studies found an increase in total *Malassezia* in patients with IBD compared to healthy controls [70]. Similarly, the microbiome of patients with CRC showed an increased level of *Malasseziomycetes* [69]. The VDR^ΔPC^ and VDR^ΔLyz^ knockouts promoted archaea with the potential to impact the sulfur and methane metabolism mechanisms of the host. Methanogenic archaea are associated with Crohn’s disease etiology [10]. The dramatic rise in *Methanofollis* after VDR deletions may predispose to IBD. The predictive functional analysis provides another angle for the relevance of this archaeal growth—competition with sulfur reduction microbes. Another abundant archaeon, *Sulfolobus* [73], is capable of sulfur metabolism [74]. Methanogens and sulfate-reducing bacteria (SRB) compete for H_2_ in the intestine [22]. Potentially, the dramatic increase in methanogens after VDR deletion is driving a concurrent drop in SRB. However, none of the most common intestinal SRB [75,76] were reported to be in lower abundance in the KO animals compared to the control mice [8]. Targeted measurement of metabolites and PCR validation of SRB are needed for future studies.

In the mammalian intestine, both archaea [73] and fungi [10] interact with the other members of the microbiome. First, the strong correlations between and within archaea and fungi suggest potential interactions within these populations and reinforce the need to consider them together in predicting host health and disease. In the VDR^ΔLyz^ mice, abundant fungi and archaea were negatively correlated with a group of benign commensals (*Bifidobacterium animalis, Bifidobacterium choerinum, Bifidobacterium pseudolongum,* and *Bacteroides acidfaciens*). This raises the possibility that they play a role in suppressing the growth of certain bacteria, which could promote a pro-inflammatory state with increased susceptibility to enteropathogenic bacteria [77]. Our previous study involving VDR^ΔLyz^ mice has shown their susceptibility to *Salmonella* infection [78]. VDR^ΔPC^ mice are also susceptible to *Salmonella* infection and intestinal inflammation [79]. The significant correlations with the virus *Lactobacillus phage phiadh* [8], which infects another commensal [80], suggest an additional layer of interaction between these communities or may reflect the decrease in *Lactobacillus johnsonii* [8]. Interestingly, the fungi and archaea of this group were also negatively correlated with the two pathogens *Clostridium perfringins* and *Bordetella psuedo hinzii.* Whether the deletion has a protective effect is unclear. Again, there is a viral analog—a positive correlation with increased levels [8] of *vibrio* phages. As bacterial phages can modulate host functionality [81], it is possible that this correlation with viruses heralds a modulation of pathogens through interactions with the archaea and fungi. At the metabolite level, there is a significant decrease in compounds formed from cysteine (N-acetylcysteine) and taurine (phenylacetyltaurine) but increases in three secondary bile acids derived from taurine (taurodeoxycholate, taurohyodeoxycholic acid, and taurochenodeoxycholate). The significant correlations among differentially abundant microbes across domains and metabolites are worthy of further exploration.

It is possible that the sex-mediated dysbiosis shown here constitutes one aspect of a multifactorial perturbation driving illnesses. The sex of an animal impacts the development and clinical presentation of gastrointestinal diseases, like IBD [82] and infections [83], in that animal. Indeed, sex has been shown to impact the mammalian mycobiome, even in the absence of pathology [84]. It is therefore unsurprising that the sexes of the VDR knockout mice modulate the impact that this deletion has on the total archaeal diversity and fungal species levels in the guts of those mice. A recent study on CRC and the microbiome showed the gender difference that *Carnobacterium maltaromaticum* boosts intestinal vitamin D production to suppress CRC in female mice [85]. Gender differences were also noticed in our previous studies on VDR regulation of the virome [8] and high-fat diet (HFD) obesity [30]. HF- fed female VDR^ΔIEC^ mice showed increased taurolithocholate 3-sulfate and taurocholenate sulfate levels. Given these findings, future vitamin D/VDR studies should continue to elucidate whether the differences seen in our study have a functional impact on the intestinal microbial community in human diseases.

There are several limitations of this study. One limitation comes from the sequencing and isolation methods. Most microbiota isolation and sequencing protocols are specially designed for identifying bacteria, which biases them away from detecting archaea and fungi [20]. Additionally, microbiome studies are intrinsically limited to establishing associations between variables rather than causality [86]. Further experiments, like the use of selective antibiotics to remove certain organisms from the intestinal niche [13], measurements like methane exhalation [87], or targeted fecal metabolomics [88], are required to evaluate vitamin or methane synthesis and to elucidate the mechanisms and physiological functions involved in the impact of a VDR deficiency.

## 5. Conclusions

**Figure 12:**
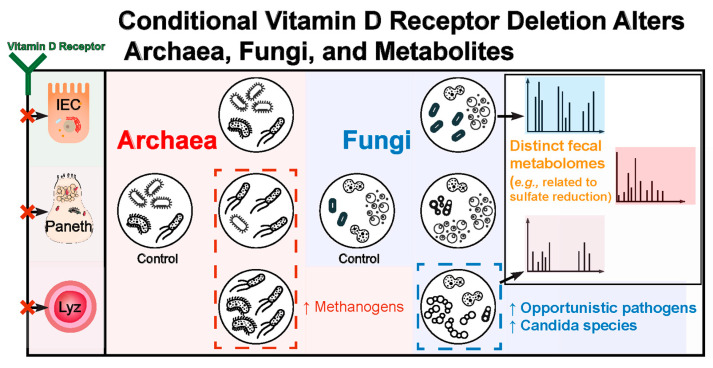
The working model of VDR deletion and altered archaea, fungi, bacteria, viruses, and metabolites.

In the current study, we report the significant correlations existed among altered archaea, fungi, bacteria, viruses, and metabolites in the VDR deficiency mice in a tissue- and gender-dependent manner. These leads to changes in the biologic functions, e.g., sulfate reduction and biosynthesis of Vitamin B12 (Figure 12). By identifying the specific fungi and archaea implicated in VDR deficiencies and the networks of microbes with which they may be interacting, we open the door for studies to elucidate how these understudied microbial populations may be contributing to the effects of a VDR deficiency. Indeed, our functional metagenomic results provide promising physiologic processes through which fungi and archaea may be driving or worsening the impact that a VDR deficiency has on the risks of certain diseases.

## Figures and Tables

**Table 1:** KEGG [61] database search looking for the presence (+) or absence (-) of a gene within the genomes of differentially abundant microbes. While the genomes of all differentially abundant microbes were searched, only the microbes with at least one of the above genes (10A) in their genome are presented.

	Sulfate Adenylyltransferase Subunit 1	Sulfate Adenylyltransferase Subunit 2	Adenosylcobyric Acid Synthase	Adenosylcobinamide-Phosphate Synthase
	K00956	K00957	K02232	K02227
*Thermococcus piezophilus*	-	-	+	+
*Bordetella pseudohinzii*	+	+	-	-
*Chroococcidiopsis thermalis*	-	-	+	+
*Sulfurovum lithotrophicum*	+	+	+	+

## Data Availability

The data will be posted in https://zenodo.org/ (accessed on 1 December 2023).

## Supplementary Materials

The following supporting information can be downloaded at: https://www.mdpi.com/article/10.3390/metabo14010032/s1, Figure S1: Fungi Correlations in Sex-Subsets; Figure S2: Archaea Correlations in Sex-Subsets; Figure S3: Differential Fungi in the KO mice; Figure S4: Sex-Differences of Fungal Dysbiosis in the VDR KO mice; Figure S5: Sex-Differences of Archaeal dysbiosis in VDR KO mice.
