# Supplementary material for: Conditional Vitamin D Receptor Deletion Induces Fungal and Archaeal Dysbiosis and Altered Metabolites
Source: Metabolites. 2024 Jan 1;14(1):32. doi: 10.3390/metabo14010032 (PMC10819266; doi:10.3390/metabo14010032)
Supplement: Supplementary file 1 [file metabolites-14-00032-s001.zip › metabolites-2780664-supplementary.pdf]

Supplementary Materials:

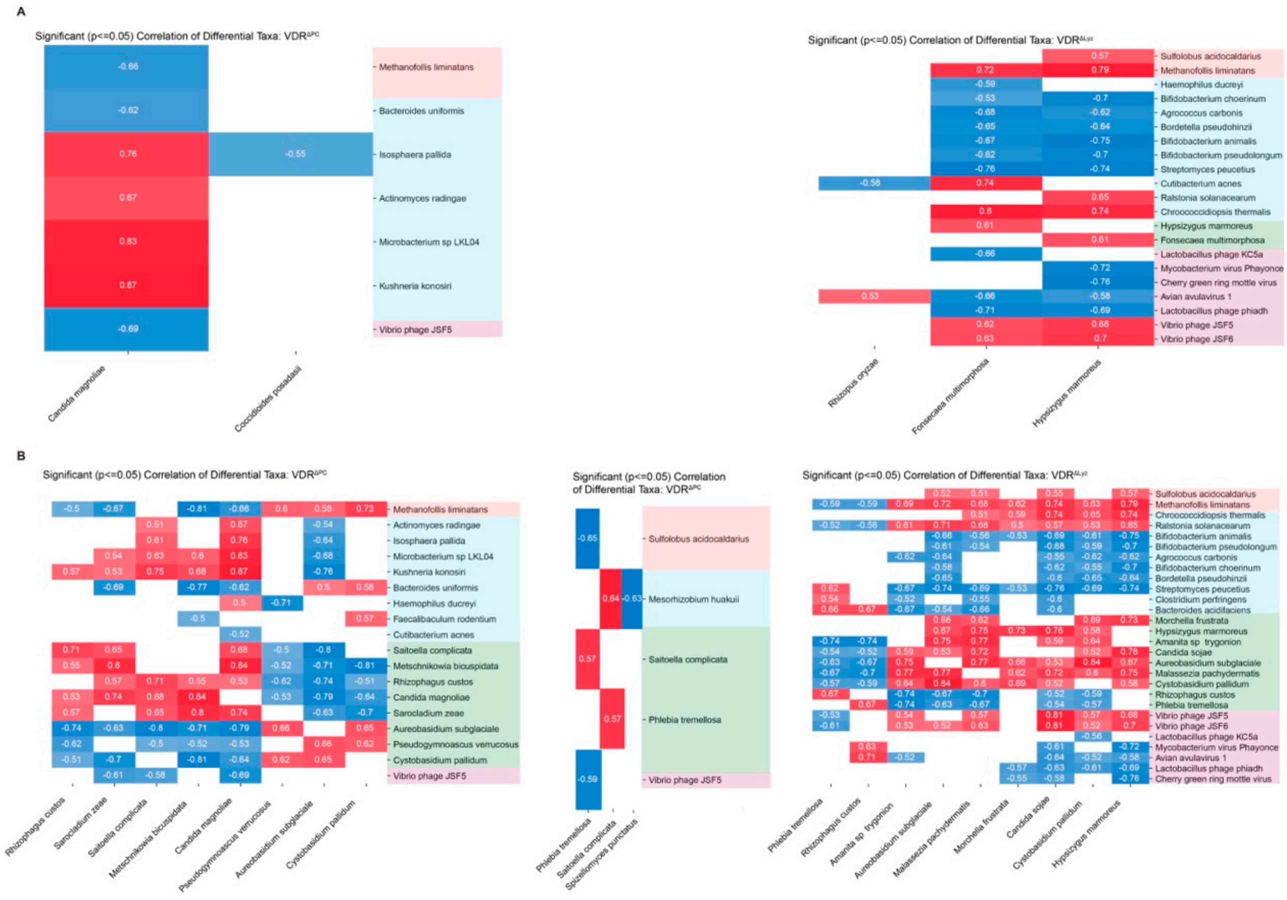

Supplemental Figure S1. Fungi Correlations in Sex-Subsets. (A) Male mice. (B) Female mice. Significant (P-value <=0.05) correlations between significantly (Q value <=0.1) differentially abundant species of *fungi* and significantly (Q-value <=0.1) differentially abundant archaea, bacteria, and viruses. The color indicates the magnitude of the correlation (blue = negative, red=positive) while a white space indicates no significant correlation. The colors of the row names indicate: peach for archaea, blue for bacteria, green for eukaryote, and purple for viruses.

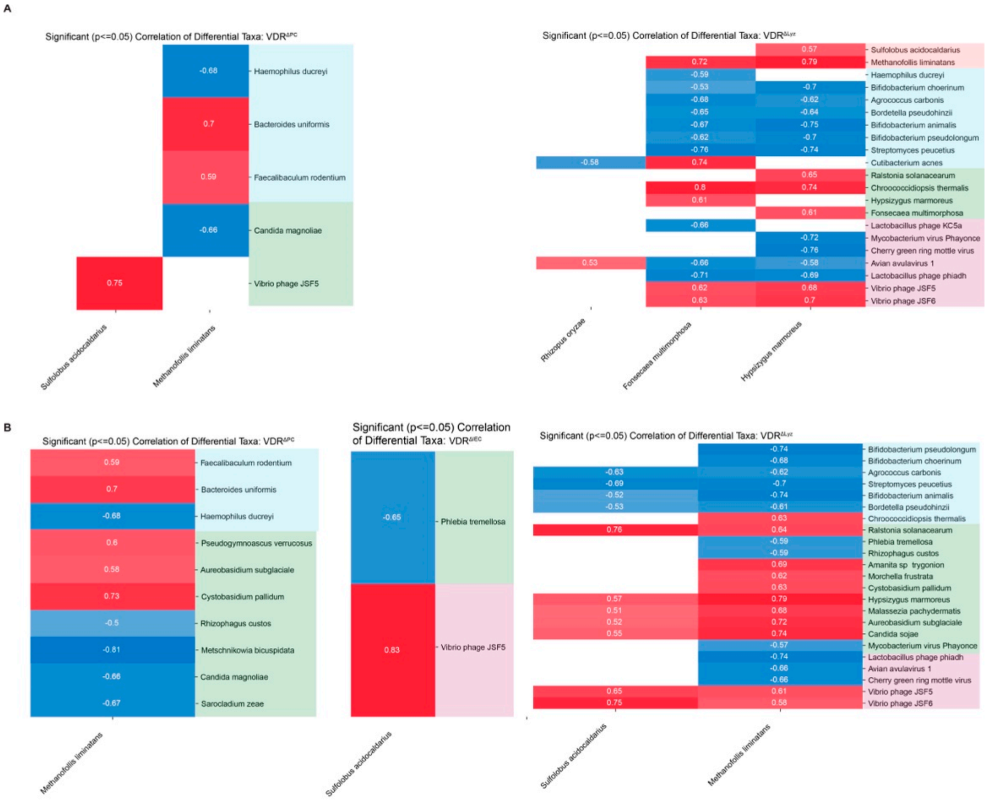

Supplemental Figure S2. Archaea Correlations in Sex-Subsets. (A) Male mice. (B) Female mice. Significant (P-value <=0.05) correlations between significantly (Q value <=0.1) differentially abundant species of archaea and significantly (Q-value <=0.1) differentially abundant bacteria, fungi, and viruses. The color indicates the magnitude of the correlation (blue = negative, red=positive) while a white space indicates no significant correlation. The color of the row names indicate: peach for archaea, blue for bacteria, green for eukaryote, and purple for viruses.

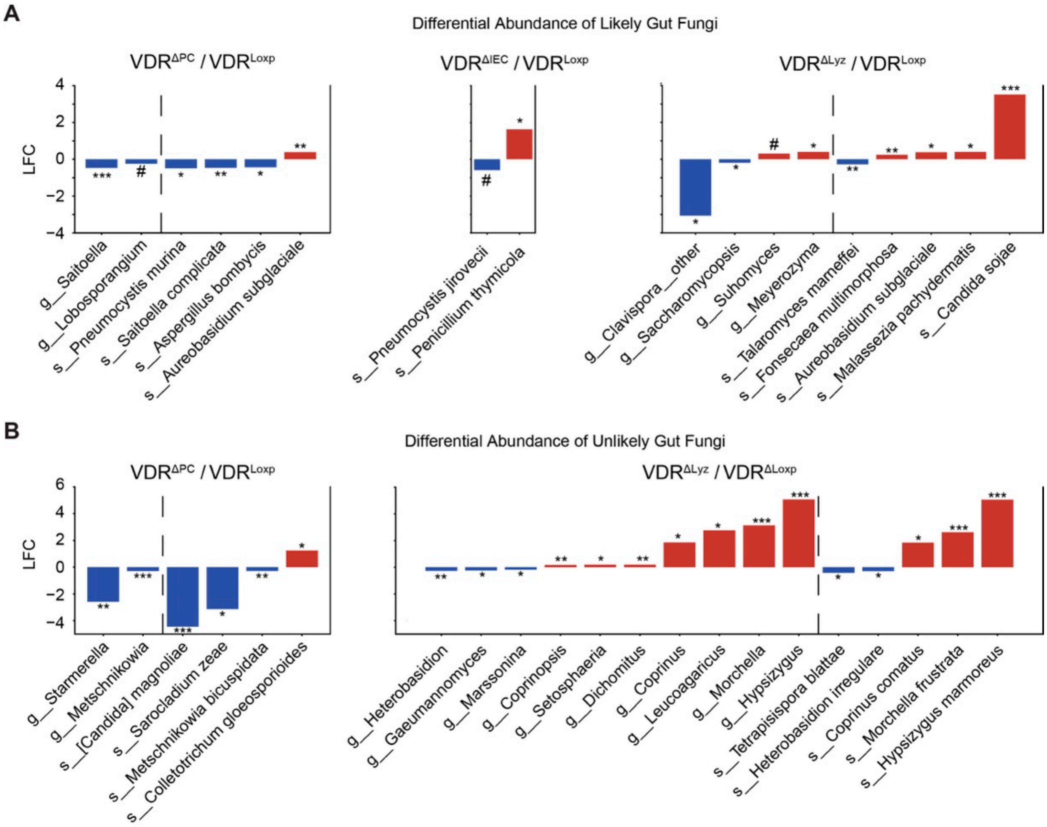

Supplemental Figure S3. Differential Fungi in the KO mice. (A) Fungi likely to be active in the gut. (B) Fungi unlikely to be active in the gut. Fungal taxa that are significantly (Q value, #<=0.1, *<=0.05, **<=0.01, ***<=0.001) differentially abundant in VDR KO mice ($VDR^{\Delta PC}$, $VDR^{\Delta IEC}$, and $VDR^{\Delta Lyz}$) relative to the control group, not sub-dividing by sex. "s_" indicates the taxa is a species and "g_" indicates it is a genus - the only two taxonomic levels studied. LFC indicates Log2 fold change and the color indicates direction of shift relative to control (blue is decreased and red is increased).

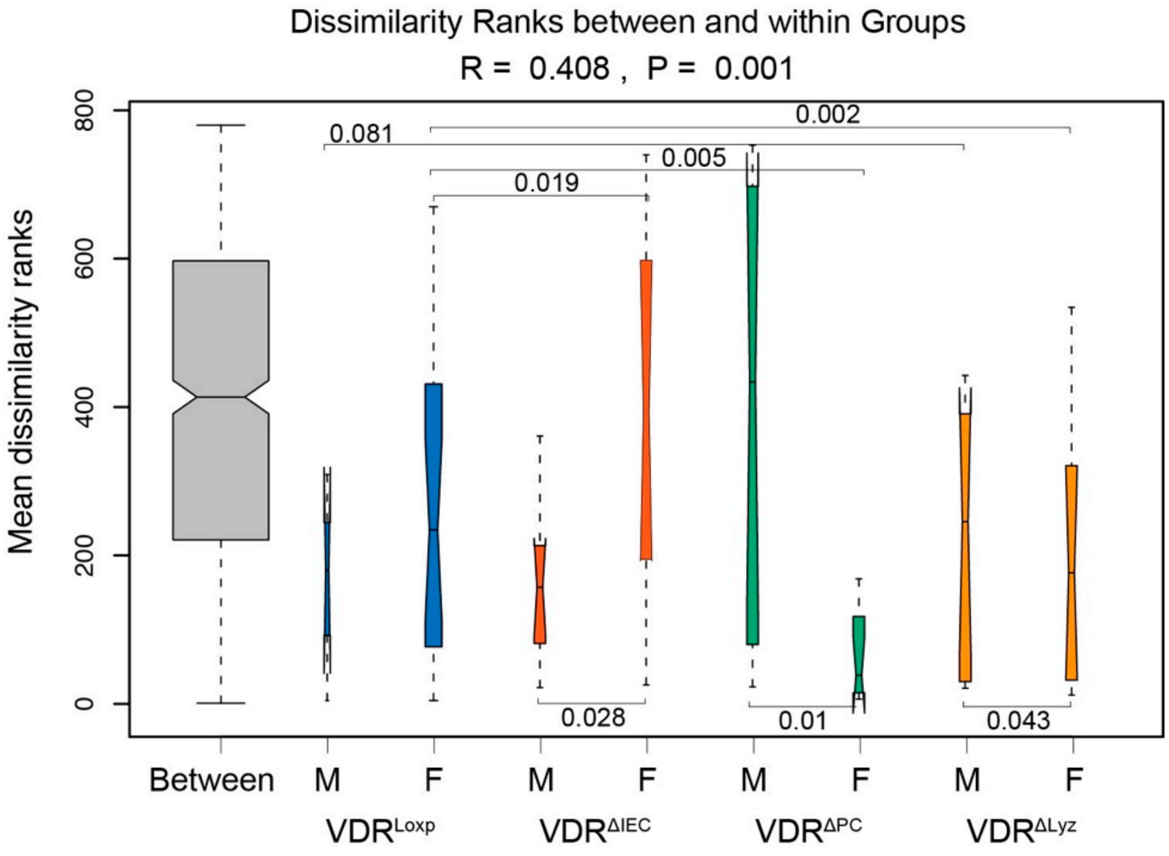

Supplemental Figure S4. Sex-Differences of Fungal Dysbiosis in the VDR KO mice. Within-group versus between-group Bray-Curtis dissimilarity metrics for fungal species in mice, split by sex. Color indicates genotype. A PERMANOVA test was applied to each to evaluate significance. The sex by genotype interaction was found to be significant (p=0.002). Presented are the p values for subsequent ANOSIM analysis on sex-subgroups that were statistically significant (p≤0.05) or near significant (p≤0.1).

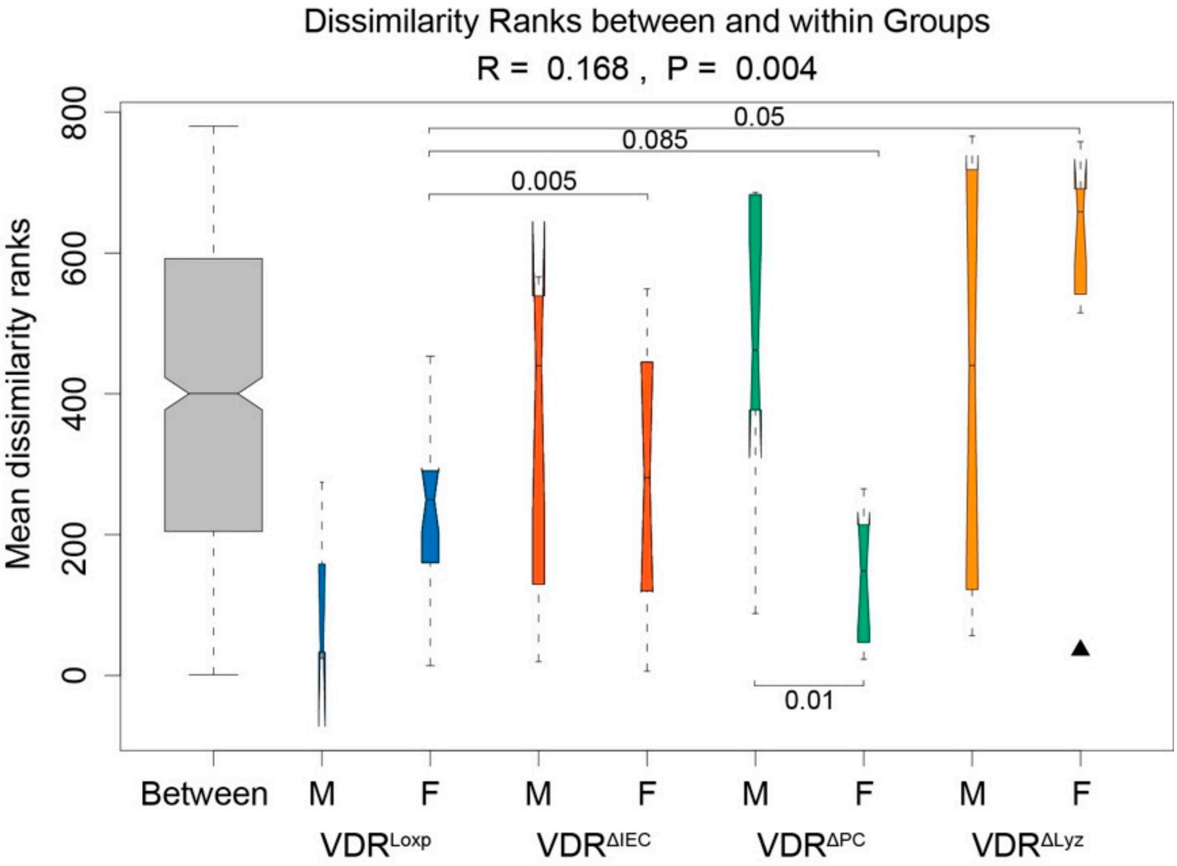

Supplemental Figure S5. Sex-Differences of Archaeal dysbiosis in VDR KO mice. Within-group versus between-group Bray-Curtis dissimilarity metrics for archaeal species in mice, split by sex. Color indicates genotype. A PERMANOVA test was applied to each to evaluate significance. The sex by genotype interaction was found to be significant (p=0.002). Presented are the p values for subsequent ANOSIM analysis on sex-subgroups that were statistically significant (p≤0.05) or marginally significant (p≤0.1).
